# Editorial: Gastrointestinal Immunity and Crosstalk With Internal Organs in Fish

**DOI:** 10.3389/fimmu.2021.734538

**Published:** 2021-09-28

**Authors:** Nan Wu, Rune Waagbø, Min Wan, Carmen G. Feijoo, Wei-Dan Jiang

**Affiliations:** ^1^Institute of Hydrobiology, Chinese Academy of Sciences, Wuhan, China; ^2^Department of Biological Sciences, Institute of Marine Research/University of Bergen, Bergen, Norway; ^3^Department of Aquaculture, College of Fisheries, Ocean University of China, Qingdao, China; ^4^Facultad de Ciencias de la Vida, Universidad Andres Bello, Santiago, Chile; ^5^Animal Nutrition Institute, Sichuan Agricultural University, Ya’an, China

**Keywords:** teleost fish, gastrointestinal immunity, crosstalk, foodborne inflammation, pathogen, vaccine

Recently, fish mucosal immunity has gradually become a hot spot in the basic study of aquaculture disease control. Mucosae is not only the physical barrier that separate teleost fish body from the environment, but it is armed with a complex network of cellular and humoral factors of innate and adaptive immunity, which work in coordination to protect them from infection or inflammation in a pathogen rich aquatic environment. Fish intestinal mucosal system is involved in the disease-control of water pathogens, as well as the induction of oral tolerance to dietary antigens and microbiota. To do so, the different immune cells that form part of the gut associated lymphocyte tissue (GALT) are distributed in the intestinal epithelial layers (IEL) and lamina propria (LP), and from there detect innocuous and harmful antigens as well as interact with the mucosal microflora.

Cytokines, chemokines and antibodies that reach blood circulation after immune response has been triggered in the gut, could stimulate other organs, such as liver, spleen and kidney. Although the local mucosal immune response could repress the infection process to a certain extent, the systemic and mucosal innate and adaptive immune cells or molecular components activated in the gut upon stimulation, will still interact with other internal organs.

It is our pleasure to present the Research Topics of gastrointestinal immunity in fish to the readers. The present journal issue is focused on fish gastrointestinal immunity and crosstalk with internal organs and collects 13 articles, including a review article and twelve original research articles from a total of 93 authors. The contributions cover research on fish gastrointestinal immune response resulted after food, pathogens, or vaccination stimulation. Also, the effects of the crosstalk between gut and other organs have been revealed from innate and adaptive point of view.

Foodborne intestinal inflammation is a major health and welfare issue that need to be considered when using plant-derived components as ingredients for fish diets, especially for carnivorous fishes. In Atlantic salmon, an intestinal transcriptomic analysis revealed that the presence of soybean meal in the diet altered the transmembrane transporter- and channel- activities and upregulated inflammation-associated genes. This coincided with an increase in different type of immune cell, especially lymphocytes in the soybean meal-fed group (Kiron et al.). To clarify the dominant anti-nutrient factor, a study on intestinal immunity after salmon feeding with marine- or plant-derived protein and lipid, showed that mucin and antimicrobial peptides were the most affected in the latter case (Sørensen et al.). As a major anti-nutritional factor, soybean-derived glycinin cause typical foodborne enteritis in grouper, while the addition of sodium butyrate decreased the inflammation by blocking MHC II-PI3K/mTOR signaling (Yin et al.).

The intestinal barrier is often challenged during pathogen invasions. Both bacteria and virus induce intestinal immune response, activating several cytokine signaling pathways. Grass carp (*Ctenopharyngodon idella*) IL22 was reported to play a pro-inflammatory role in both intestinal mucosal and systemic immunity, and IL22^+^ cells were found to be induced during a bacterial challenge with *Flavobacterium columnare* (Yang et al.). On the other hand, after grass carp challenge with grass carp reovirus (GCRV), the progression of intestinal disorder paralleled a sharply increased gene expression of protein phosphatase PP1 (PPP1R3G) in both intestine and head kidney, indicating that fish intestine could be a frontline of innate immune response (Hu et al.). In addition, for subtle regulating of fish gastrointestinal immune gene expression, non-coding RNA regulatory networks were revealed by transcriptomic analysis. At post-transcriptional level, both lncRNA-miRNA-mRNA and ceRNA networks played a regulatory role in the intestine upon *Edwardsiella tarda* infection of marine fish, such as black rockfish (*Sebastes schlegilii*) and olive flounder (*Paralichthys olivaceus*) (Cao et al., Xiu et al.).

To control disastrous infection of pathogens at the frontline, mucosal vaccines are currently being explored as an effective tool, along with the recent achievement in fish mucosal immunology. Oral administration of a vaccine formulated with LamB antigen, obtained from duckweed (*Wolffia globosa*), was used to generate antibodies to prevent Vibriosis infection in zebrafish (*Danio rerio*) (Heenatigala et al.). Another paper demonstrated that an immersion vaccine induced highest IgM level in the intestine compared to gill and skin tissues in carp (*Carassius auratus*) and grouper (*Epinephelus coioides*) (Gong et al.). During mucosal immunization, B cell responses, evidenced as changes in immunoglobulins (IgM, IgT and IgD) levels, as well as T cell responses reflected by increased CD8 and CD4 level (Munoz-Atienza et al.), were analyzed in mucosal and non-mucosal immune organs. Yet, different effects were detected depending on the administration routes used, including oral and nasal vaccination, anal intubation and immersion vaccination in fish (Munoz-Atienza et al.).

Regarding the crosstalk between gut and other organs in fish gastrointestinal immunity, the papers included in this issue showed the participation of the liver, kidney and spleen. During *Vibrio harveyi* infection, the gut-liver axis was affected, since intestinal inflammation and dysbacteriosis were observed concomitantly with the appearance of hepatic nodules, besides the typical symptoms in the heart and kidney (Deng et al.). Head kidney macrophages of Atlantic salmon fed with a fish meal and fish oil diet, showed the highest phagocytic activity compared to fish fed with diets based on plant-derived protein and lipid (Sørensen et al.). Meanwhile, short chain fatty acids (SCFAs), which are products of microbial fermentation of dietary fiber in the gut, promoted bacterial clearance by head kidney macrophages in turbot (*Scophthalmus maximus* L.) (Zhang et al.). On the other hand, a fish meal and fish oil diet, as well as a fish meal and rapeseed oil diet induced the presence of the highest amount of lymphocytes in head kidney of Atlantic salmon (Heenatigala et al.). During immersion vaccination in carp and grouper, the existence of a hindgut-liver-spleen immune synergy was proposed based on immunoglobulin gene expressions (Gong et al.).

Overall, advances in the research of gastrointestinal immunity and its associated inter-organ crosstalk included in this issue provide novel and central data to improve our understanding of mucosal immune barrier function in teleost fish. New insights related to intestinal mucosal immune regulation are key aspects when designing better diets formulations and new mucosal vaccines, as well as genetically facilitated infection control and prevention. Eventually, deepening knowledge of the regulation of intestinal immune homeostasis will enhance fish health and further guarantee an efficient growth performance in a long-term perspective.

We hope and expect the aquaculture research community will find this collection of articles within the gastrointestinal immunity topic informative and inspiring. As editors, we would like to thank the authors for their interesting contributions, as well as express our gratitude to all referees for their careful evaluation of the papers. Many thanks to all the colleagues who responded to this call, but whose interests could not be accommodated within the confines of this research topic. Finally, we extend our sincere appreciation to *Frontiers in Immunology* for supporting this exciting Research Topic.

## Author Contributions

NW prepared the draft editorial. RW, MW, CF, and W-DJ revised the manuscript. All authors contributed to the article and approved the submitted version.

## Funding

This work was funded by the grant from National Natural Science Foundation of China (31872592) to NW.

## Conflict of Interest

The authors declare that the research was conducted in the absence of any commercial or financial relationships that could be construed as a potential conflict of interest.

## Publisher’s Note

All claims expressed in this article are solely those of the authors and do not necessarily represent those of their affiliated organizations, or those of the publisher, the editors and the reviewers. Any product that may be evaluated in this article, or claim that may be made by its manufacturer, is not guaranteed or endorsed by the publisher.

